# Promoting public health through nutrition labeling - a study in Brazil

**DOI:** 10.1186/s13690-016-0160-x

**Published:** 2016-11-14

**Authors:** Sônia Maria Fernandes da Costa Souza, Kenio Costa Lima, Maria do Socorro Costa Feitosa Alves

**Affiliations:** Federal University of Rio Grande do Norte (UFRN), Natal, RN Brazil

**Keywords:** Nutritional labeling, Nutrition, Public health

## Abstract

**Background:**

Food and nutrition education allows individuals to build knowledge and values, reframe their food practices, and develop strategies for a healthy diet. Food choices within the diet represent a determinant of individual health status. Regardless of the food quality, the consumption of calorie-dense foods does not promote better health conditions for the population and can worsen emerging health problems. The present study aimed to describe and analyze the effectiveness of educational activities related to nutrition information for enabling healthy food choices, as a tool to promote public health.

**Methods:**

To describe and analyze the effectiveness of an educational intervention regarding nutrition labeling as a tool to promote healthy food choices, 702 individuals were enrolled in the present quasi-experimental study. The Wilcoxon and McNemar tests were used to compare the pre- and post-intervention data, and a *p* value <0.05 was considered statistically significant.

**Results:**

Of the 702 participants (mean age, 26.6 years), 17.4 % were male, and 82.6 % were female. The education level was high school for 53.2 % of the participants. The mean income was R$ 1969.54 (about 500 USD). In the pre-test, 55.8 % of the respondents reported consulting the nutrition information provided on packaged foods. At the post-test, 72.0 % of respondents reported consulting this information (*p* < 0.001; Table 1). However, the change in the response regarding the purchase of packaged products was borderline significant.

**Conclusions:**

The results indicate that the intervention was feasible and acceptable and improved knowledge regarding the role of nutrition labeling in promoting healthy eating. These results support the importance of an educational intervention to reinforce healthy food choices.

## Background

Food and nutrition education allows individuals to build knowledge and values, reframe their food practices, and develop strategies for a healthy diet [1]. Food choices within the diet represent a determinant of individual health status. Regardless of the food quality, the consumption of calorie-dense foods does not promote better health conditions for the population and can worsen emerging health problems [2].

The consumption of processed foods has become increasingly common worldwide; these foods are generally characterized by energy density and high sugar, fat, salt, and sodium content [3]. Diets containing processed foods in addition to physical inactivity are associated with chronic diseases such as diabetes, hypertension, and obesity [4, 5].

Obesity is reaching epidemic proportions, more than doubling worldwide since 1980 [3]. The most common consequences of overweight and obesity are chronic non-communicable diseases, particularly cardiovascular disease, which is the leading cause of death globally as well as in Brazil. Furthermore, studies indicate that obesity and diabetes are often associated with high blood pressure [6, 7].

On an international basis, food-related guidelines, such as the North American Dietary Guidelines for Americans, 2010, and associations, such as the American Heart Association, recommend eating foods low in fat, calories, and carbohydrates [8, 9]. In 2005, the World Action on Salt and Health (WASH) was created with support from the World Health Organization (WHO) as a global initiative to reduce salt consumption to improve population health in different countries. The WASH encourages multinational food companies to reduce the salt content of its products and highlights the need to implement comprehensive strategies by governments around the world for the population to reduce salt intake [10].

The Global Strategy on Diet, Physical Activity and Health, adopted at the 57th Assembly of the WHO 2004, argues that the provision of adequate and understandable information about nutritional content, which does not mislead the consumer, can promote health and reduce the risk of food and nutrition-related diseases [11]. The labeling of food should be used as a nutrition education tool for the population, to guide consumers regarding the quality and quantity of the nutritional constituents of products and to promote appropriate food choices [12, 13].

In Brazil, the Collegiate Board-ANVISA n° 359/2003 [14] and n° 360/2003 [15] resolutions regarding Mandatory Nutritional Labeling reinforce recommendations that nutrition labeling helps consumers in their choices and to combat chronic diseases [16], particularly because lifestyle interventions can effectively reduce the risk of chronic diseases [17, 18].

The present study aimed to describe and analyze the effectiveness of educational activities related to nutrition information for enabling healthy food choices [16], as a tool to promote public health.

## Methods

High school or undergraduate students aged ≥18 years were eligible. The sample size was determined based on a pilot study conducted with 118 students that aimed to determine if they consulted nutrition information on labeling before choosing foods; the difference between the frequencies observed in the pre- (69.0 %) and post- (78.0 %) tests was 8.5 %. Therefore, the estimated required sample size was 800 students based on an alpha error of 5 % and a beta error of 20 %.

The study was approved by the Ethics Committee of the Federal University of Rio Grande do Norte, Rio Grande do Norte State, Brazil (opinion number 034/2010).

The intervention occurred over a period of 30 days, with a pre-test and a post-test.

### Pre-test

During the pre-test, the participants were asked to complete a self-administered structured questionnaire of 22 questions; the questions with >30 % divergence in the original version had been reformulated in the final version. In addition to other questions, the participants were asked if they purchased packaged foods and if they checked the nutrition information before purchase. In addition, the participants were asked to identify whether a food was healthy using a traffic light system and based on a table containing nutrition information for product examples (e.g., a product that had 400 mg of sodium per 100 g of product).

Then, the participants were provided a folder of educational material to promote the understanding of nutrition information and its use for healthier food choices. The material was developed using the Board Resolutions Collegiate Agência Nacional de Vigilância Sanitária (ANVISA) numbers 259/2002 [19], 359/2003 [14], 360/2003 [15], 24/2010 [20], and 54/2012 [21]; the Food Guide for the Brazilian population [22]; and the report entitled *Comprehension and use of UK nutrition signpost labelling schemes* [23]. The material was refined during a pre-pilot study with 158 consumers, after which it was submitted to and recorded by the National Library in Rio de Janeiro as an original literary work in Brazil (number 574,806). Thereafter, it was reproduced and used in this intervention study.

The participants were also involved in a 50-min dialogue and exposure program that was developed by the researcher and addressed nutrition labeling legislation; importance of nutrition information for choosing healthier foods and the relation with chronic noncommunicable diseases; use of a “nutrition traffic light” for added sugar, saturated fat, sodium, and fiber content (green indicates appropriate levels, red indicates inappropriate levels); and recommended daily allowances, with respect to the list of ingredients with nutrition information and as a parameter to help select healthier foods.

### Post-test

Thirty days after the pre-test, the same participants completed the questionnaire again.

Dichotomous responses were compared using the McNemar test, and ordinal responses were compared using the Wilcoxon test. Statistical significance was set at *p* < 0.05.

## Results

Of the 702 participants (mean age, 26.6 years), 17.4 % were male, and 82.6 % were female. The education level was high school for 53.2 % of the participants. The mean income was R$ 1969.54 (about 500 USD).

Based on the calculated sample size, the included sample size was <13 % smaller, and there was <5 % loss to follow-up at the post-test, after consideration of participants who did not answer some of the questions that were in the pre- or post-test or both phases.

In the pre-test, 55.8 % of the respondents reported consulting the nutrition information provided on packaged foods. At the post-test, 72.0 % of respondents reported consulting this information (*p* < 0.001; Table 1). However, the change in the response regarding the purchase of packaged products was borderline significant.

**Table 1:** Comparison of responses to questions relating to behaviors and knowledge between the pre- and post-test

Questions	Post-test	Pre-test	Post-test	Pre-test	*p*
Do you purchase packaged foods?	Yes (%)	No (%)	Yes (%)	No (%)	0.072
Do you often read the nutrition information before purchase?	690 (98.3)	12 (1.7)	690 (98.3)	12 (1.7)	<0.001
Does the nutrition information help you choose healthier foods?	391 (55.9)	309 (44.1)	391 (55.9)	309 (44.1)	1.000
Should the nutrition information be used in supermarkets and other commercial food establishments as a nutrition education strategy?	698 (99.7)	2 (0.3)	698 (99.7)	2 (0.3)	1.000
Did the education material promote the use and understanding of nutrition information?	694 (99.1)	6 (0.9)	688 (98.8)	8 (1.2)	0.796
Does the nutrition traffic light with the maximum and minimum values for recommended consumption of sugar, fats, sodium, and fiber promote the use and understanding of nutrition information?	695 (99.1)	6 (0.9)	687 (98.6)	10 (1.4)	0.424

Analyses were conducted using the McNemar test

When asked if the nutrition information helps in choosing healthy foods or whether nutritional guidance should be used in supermarkets or other commercial food establishments, 100 % of respondents answered yes, both pre-test and post-test. When asked if the use of a nutrition traffic light with the maximum and minimum recommended consumption of sugar, fats, sodium, and fiber promotes the understanding of nutrition labeling, the responses were significant (Tables 1, 2, 3 and 4).

**Table 2:** Comparison of responses to questions regarding recommended consumption of key nutrients between the pre- and post-test

Questions	Post-test	Pre-test	Post-test	Pre-test	p
The sugar intake should be?	Low (%)	High (%)	Low (%)	High (%)	0.124
The consumption of saturated fats and trans must be?	675 (96.8)	22 (3.2)	675 (96.8)	22 (3.2)	0.296
Sodium intake should be?	675 (97.7)	16 (2.3)	675 (97.7)	16 (2.3)	0.003
Fiber consumption should be?	662 (95.9)	28 (4.1)	662 (95.9)	28 (4.1)	0.401

Analyses were conducted using the McNemar test

**Table 3:** Statistical representation of the comparison between the responses in the first and second steps of the intervention - McNemar test. Natal, Rio Grande do Norte, Brazil

Questions	Pre-test		Post-test		*P*
	Red (%)	Green (%)	Red (%)	Green (%)	
What the traffic light color of the nutrient after to check the Nutrition Information table?	563 (81.8)	125 (18.2)	596 (87)	89 (13)	0.005

**Table 4:** Statistical representation of the comparison between the answers in the first and second steps of the intervention - Wilcoxon test. Natal, Rio Grande do Norte, Brazil

Question	Pre-test			Post-test			*P*
	Nothing (%)	Partial (%)	All (%)	Nothing (%)	Partial (%)	All (%)	
What was understood from nutritional guidance about the Nutrition Information?	-	271 (45.1)	330 (54.9)	3 (0.5)	294 (48.4)	310 (51.1)	0.003

The participants positively evaluated the education material provided in the folder, the orientation during the program, and the importance of knowledge of the recommended daily values (Table 5).

**Table 5:** Comparison of responses to questions regarding the importance of nutrition information between the pre- and post-test

Question	Pre-test				Post-test				*P*
	Unimportant (%)	Few important (%)	Important (%)	Very important (%)	Unimportant (%)	Few important (%)	Important (%)	Very important (%)	
Evaluation of nutritional guidance received about nutritional information	-	5 (0.7)	172 (24.6)	521 (74.6)	-	6 (0.9)	246 (35.2)	447 (63.9)	<0.001
What do you think about the nutrition labeling legislation?	-	6 (0.9)	276 (39.5)	416 (59.6)	-	11 (1.6)	335 (47.8)	355 (50.6)	<0.001
It is important to know the relationship of nutrition information to a healthier food?	-	-	130 (18.6)	568 (81.4)	1 (0.2)	3 (0.4)	188 (26.9)	508 (72.7)	<0.001
It is important to know the recommended daily consumption values?	-	4 (0.6)	211 (30.2)	484 (69.2)	-	7 (1)	263 (37.6)	429 (61.4)	<0.001
It is important to know the daily consumption values and nutritional information?	-	-	212 (30.3)	488 (69.7)	-	9 (1.3)	265 (37.8)	427 (60.9)	<0.001
It is important to know the maximum and minimum consumption values per serving of sugar. fats. sodium and fiber?	1 (0.1)	3 (0.4)	143 (20.5)	551 (78.9)	3 (0.4)	8 (1.1)	168 (24)	521 (74.5)	0.006
What to you think about the Folder?	-	1 (0.1)	268 (38.2)	432 (61.6)	4 (0.6)	8 (1.1)	305 (43.7)	381 (54.6)	<0.001

## Discussion

The majority of the study respondents were women, reported purchasing packaged foods, and recognized the relevance of the information provided by nutrition labeling, which indicates that scientific information can be translated to a form that strengthens the ability of students to choose healthy foods [24].

The findings identified a change in attitudes regarding the understanding and use of nutrition information between the pre-test and post-test. A previous study that addressed the relationship between food characteristics and eating behaviors reported that food preferences change as a result of experiences and learning [25].

During the pre-test phase, the participants were provided the folder of educational information to read and consult, as needed, until the post-test phase. At the post-test, the respondents reported using the information and that they were motivated by the material, supporting the results of another study in which consumers were receptive to the information and guidance provided by qualified professionals in supermarkets and schools for nutrition labeling [26]. In addition, there was a substantially greater tendency for participants to read labels before food purchases following an intervention in another study [27]. Collectively, these results demonstrate the importance of customized educational programs to promote healthy lifestyles [28].

The nutrition traffic light system helped to identify appropriate and inappropriate sugar, fat, sodium, and fiber content and significantly improved healthy food choices. There is substantial global evidence indicating that interpretive labels (using graphics, symbols, or colors) are better understood than the traditional numerical nutrition labels [29]. Similarly, a review conducted by the European Heart Network in the United States and Europe found that consumers have difficulties understanding nutrition information, particularly regarding technical terms and numeric information requiring calculations, as well as understanding the roles that different nutrients play in their diets [30]. Thus, in 2006, the Food Standards Agency (FSA) in the UK recommended that retailers and food manufacturers include a traffic light system that uses a color code for the fat, saturated fat, sugar, and salt content (red, high levels; orange, medium levels; green, low levels) on the front panel of the packaging of various products [19]. This information allows a quick assessment of the relative merits of several similar products [31]. Furthermore, the US Centers for Disease Control and Prevention, in conjunction with the Food and Drug Administration (FDA), issued a final report in 2012 from a committee of experts that convened to create recommendations for the nutrition information on the front of the packaging [32].

Compared with that at the pre-test, fewer participants reported fully understanding the nutritional information after the intervention. However, the number of participants who reported partial comprehension of the nutritional information increased. This apparent contradiction is explained by the collection of self-reported knowledge; before the intervention, most participants believed that they fully understood the nutritional information, when they likely did not really understand it. However, after the intervention, the number of participants who actually fully understood the nutritional information increased, as verified by the change in attitudes about food choices.

Interventions directed at a variety of cultural, environmental, physical, social, and personal factors can positively influence healthy food consumption [2, 5]. Therefore, complementary and specific nutrition education interventions might be necessary to influence consumer behaviors and attitudes to choose healthy foods in supermarkets and restaurants [33].

Because participants are likely to return to previous behaviors within 5 years without maintenance of education [34], maintenance programs for health education are essential for people to fully understand the information and continue to practice the skills needed for healthy lifestyles [35].

## Conclusions

The intervention described in this study was feasible and acceptable, improving the knowledge of the participants and providing tools to promote healthy food choices.

Because definitive causal inferences about an educational intervention cannot be made, a quasi-experimental study provides information about the effects of an educational intervention in a specific population.

In addition, there are certain limitations to the present study, due to the lack of other studies that have used similar methods and theoretical frameworks. More evidence is needed to substantiate the impact of this intervention, by addressing the influence of attitudes and buying behaviors of consumers.

## Abbreviations

ANVISA: Agência Nacional de Vigilância Sanitária (National Agency for Sanitary surveillance)
FDA: Food and Drug Administration
FSA: Food Standards Agency
WASH: World Action on Salt and Health
WHO: World Health Organization
